# Role of sea-ice initialization in climate predictability over the Weddell Sea

**DOI:** 10.1038/s41598-019-39421-w

**Published:** 2019-02-25

**Authors:** Yushi Morioka, Takeshi Doi, Doroteaciro Iovino, Simona Masina, Swadhin K. Behera

**Affiliations:** 1 Application Laboratory, JAMSTEC, Yokohama, Japan; 2 Fondazione Centro Euro-Mediterraneo sui Cambiamenti Climatici (CMCC), Bologna, Italy

## Abstract

Potential impact of sea-ice initialization on the interannual climate predictability over the Weddell Sea is investigated using a coupled general circulation model. Climate variability in the Weddell Sea is generally believed to have association with remote forcing such as El Niño-Southern Oscillation and the Southern Annual Mode. However, sea-ice variability in the Weddell Sea has been recently suggested to play additional roles in modulating local atmospheric variability through changes in surface air temperature and near-surface baroclinicity. Reforecast experiments from September 1st, in which the model’s sea-surface temperature (SST) and sea-ice concentration (SIC) are initialized with observations using nudging schemes, show improvements in predicting the observed SIC anomalies in the Weddell Sea up to four months ahead, compared to the other experiments in which only the model’s SST is initialized. During austral spring (Oct–Dec) of lower-than-normal sea-ice years in the Weddell Sea, reforecast experiments with the SST and SIC initializations reasonably predict high surface air temperature anomalies in the Weddell Sea and high sea-level pressure anomalies over the Atlantic sector of the Southern Ocean. These results suggest that accurate initialization of sea-ice conditions during austral winter is necessary for skillful prediction of climate variability over the Weddell Sea during austral spring.

## Introduction

Ocean and climate systems in the Weddell Sea, located east of the Antarctic Peninsula (60°W-0°), are greatly influenced by sea-ice variability in the region across different timescales. On seasonal timescales, an increase in sea-ice extent (SIE) during austral autumn and winter inhibits air-sea interaction at the ocean surface and plays a critical role in generating cold and saline water that contributes to formation of the Antarctic Bottom Water^1–3^. Although the SIE over the Weddell Sea does not present a significant trend^4^, it undergoes distinct interannual variability with two peaks observed during austral autumn and spring^5,6^, probably associated with a semiannual cycle of the internal atmospheric variability in the Southern Hemisphere^7,8^.

The interannual variability of sea-ice concentration (SIC) in the Weddell Sea has so far been considered as a response to the atmospheric forcing^5^. Recently, the interannual sea-ice variability in the Weddell Sea has been suggested to play additional roles in modulating the atmospheric variability in the Atlantic sector of the Southern Ocean^6^. During the sea-ice melting phase of Oct–Dec, lower-than-normal SIC anomalies in the Weddell Sea receive more incoming solar radiation due to decreased surface albedo with a consequent enhancement of the surface air temperature (SAT) in the Weddell Sea. The high SAT anomalies act to weaken meridional temperature gradient and near-surface baroclinicity to the north of the Weddell Sea, which provides favorable conditions for reducing storm track activity and maintaining anticyclonic atmospheric anomalies in the Atlantic sector of the Southern Ocean. Although it does not play a role as strong as the other dominant remote forcing of El Niño-Southern Oscillation (ENSO) and the Southern Annular Mode (SAM), realistic representation of the SIC variability in the Weddell Sea is required for skillful prediction of the atmospheric variability there.

Many studies have reported that interannual SIC variation in the Weddell Sea is closely linked with ENSO^9–12^. The sea ice in the Weddell Sea generally tends to expand further north during El Niño years (vice versa in La Niña years). The associated cyclonic atmospheric anomalies developed northeast of the Weddell Sea aid to advect cold wind anomalies equatorward from the Antarctica^11,12^. In such a scenario, precise simulation of atmospheric teleconnection from ENSO, called the Pacific-South American pattern (PSA)^13^, may be necessary for skillful prediction of the sea-ice variability in the Weddell Sea. However, the sea ice in the Weddell Sea experiences interannual variability even in the absence of ENSO. That happens due to local influences arising in the Weddell Gyre circulation^5^ and the other atmospheric forcing such as the SAM^12,14,15^. Considering that sea ice plays a key role in surface heat flux exchange between the ocean and atmosphere, accurate initialization of sea-ice conditions, such as the SIC, may help skillful prediction of sea-ice variability in the Weddell Sea region and hence the local atmospheric variability.

Along this line, this study aims to examine the predictability of the SIC in the Weddell Sea by conducting two reforecast experiments using a coupled general circulation model (CGCM): one experiment adopts SST initialization in which the model’s SST is nudged to the observed SST using surface heat flux, while the other additionally employs SIC initialization in which the model’s SIC is restored to the observed SIC. Differences in the two reforecast experiments highlight potential impacts of SIC initialization on prediction of the SIC variability and the overlying atmospheric variability. Since previous studies have mainly focused on prediction skills of the global average of sea-ice extent in the Antarctica^16,17^, this study would further explore predictability of the regional SIC variability and the associated atmospheric variability over the Weddell Sea.

## Results

### Prediction skills of sea-ice concentration in the Weddell Sea

**Figure 1:**
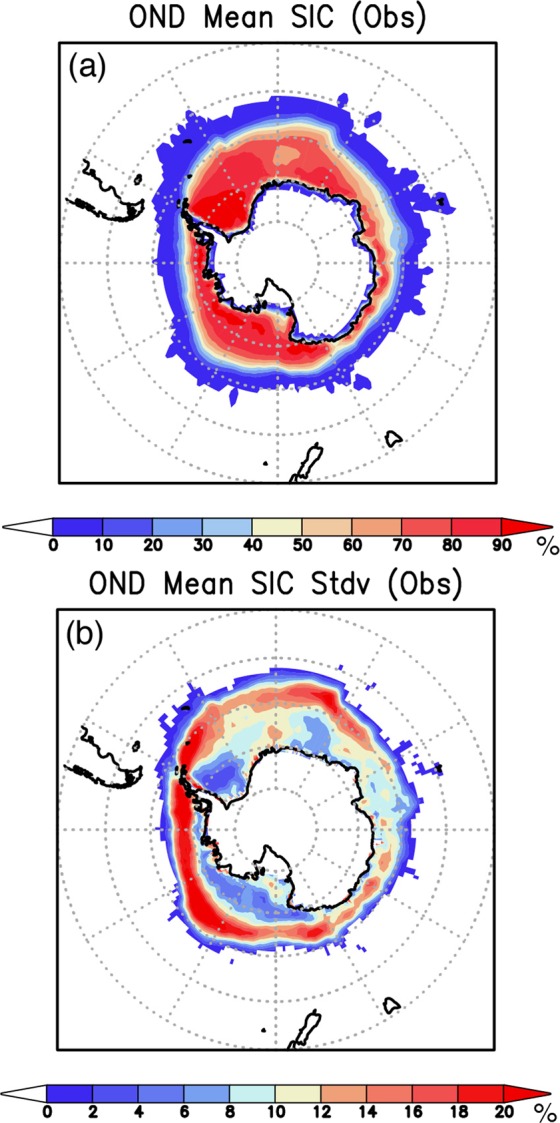
(**a**) Climatological mean sea-ice concentration (SIC, color in %) observed during Oct–Dec of 1983–2016 from the OISST v2 dataset. (**b**) Same as in (**a**), but for the standard deviation (Stdv, color in %). The maps were generated using Grid Analysis and Display System (GrADS) Version 2.1.a3 (http://cola.gmu.edu/grads/downloads.php).

Climatologically, the Antarctic sea ice heads to its maximum extent in September, then retreats until reaching its minimum in February. During the early retreat season of Oct–Dec, the SIC in the Weddell Sea and the Ross Sea remains high above 50% (Fig. 1a). The interannual variability, the standard deviation of the SIC, shows large values near the sea-ice edge (Fig. 1b), highlighting large fluctuations of sea-ice edge interacted with ocean and atmosphere^18^. In particular, regional seas in the West Antarctic (Ross Sea, Amundsen Sea, Bellingshausen Sea, and Weddell Sea) tend to undergo larger interannual variability of the SIC than that in the East Antarctic^18^.

**Figure 2:**
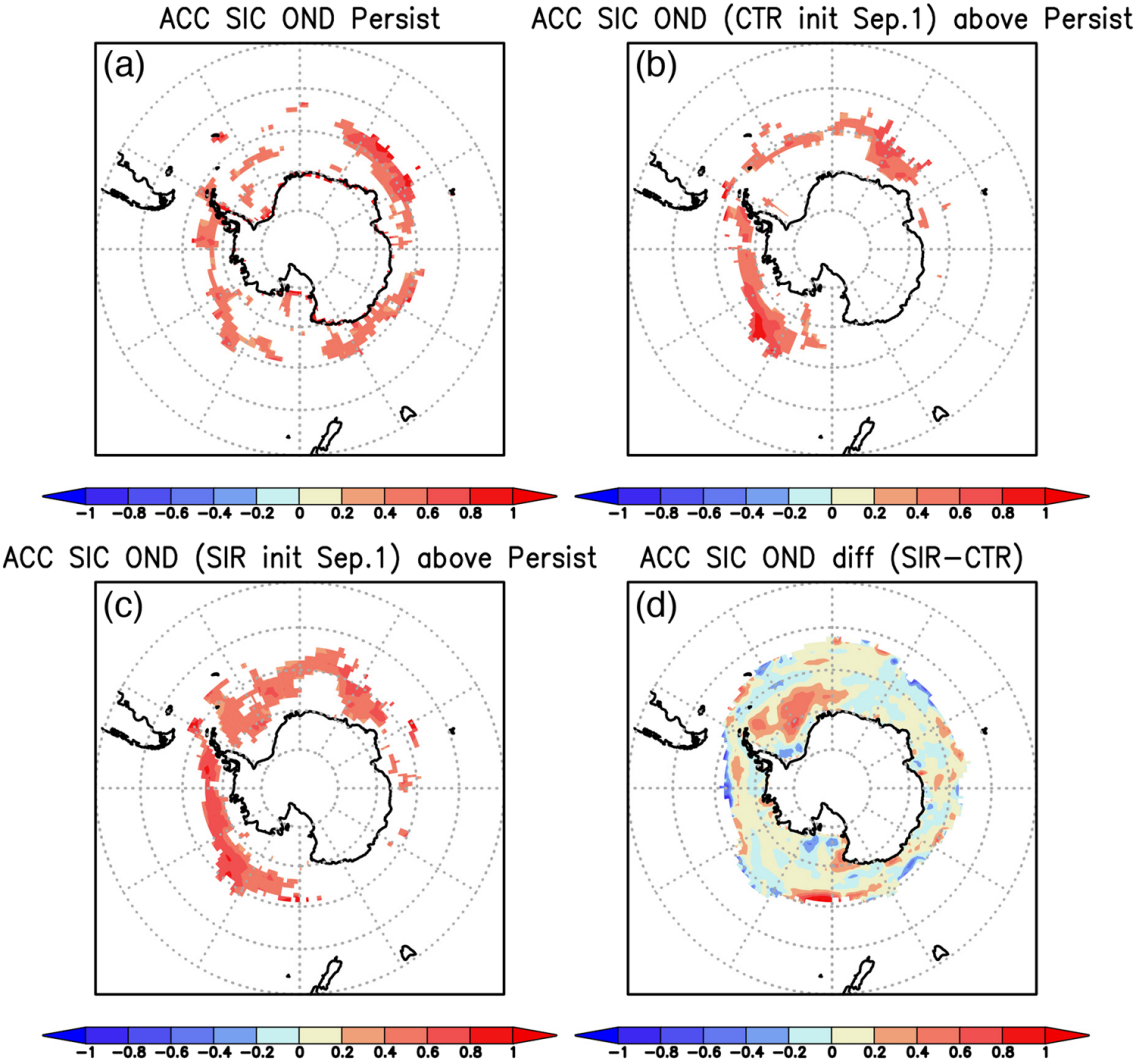
(**a**) Anomaly correlation coefficient (ACC; Persistence) of the observed sea-ice concentration (SIC) anomalies between Jun-Aug and Oct–Dec seasons. Statistically significant ACCs at 95% confidence level of a two-tailed Student’s *t* test are shaded. (**b**) ACC of the Oct–Dec mean SIC anomalies between the observation and the CTR reforecast results from September 1st. Positive ACCs which exceed persistence values in (**a**) and are statistically significant at 95% confidence level of a two-tailed Student’s *t*-test are shaded. (**c**) Same as in (**b**), but from the SIR reforecast results from September 1st. (**d**) Differences in the ACCs between the SIR and CTR reforecast results. The maps were generated using Grid Analysis and Display System (GrADS) Version 2.1.a3 (http://cola.gmu.edu/grads/downloads.php).

The prediction skills of the Antarctic SIC are evaluated using the persistence values and anomaly correlation coefficients (ACCs) between the observed SIC and the predicted SIC in the two reforecast experiments (Fig. 2). This allows us to specifically highlight some of regions in the Antarctic seas that show improvements in the SIC predictability. The persistence values show moderately high skills in predicting the Antarctic SIC anomalies during Oct–Dec, but the prediction skills in the Weddell Sea are very limited (Fig. 2a). On the other hand, the CTR experiment, even with the SST initialization only, demonstrates some improvements in the prediction skills of the SIC anomalies in the West Antarctica compared to the persistence values (Fig. 2b). Also, in the SIR experiment where the model’s SIC is additionally initialized in the CTR experiment, the ACC shows much improvement particularly in the Weddell Sea (Fig. 2c). This can be clearly seen in differences of the ACCs between the SIR and CTR reforecast results (Fig. 2d).

**Figure 3:**
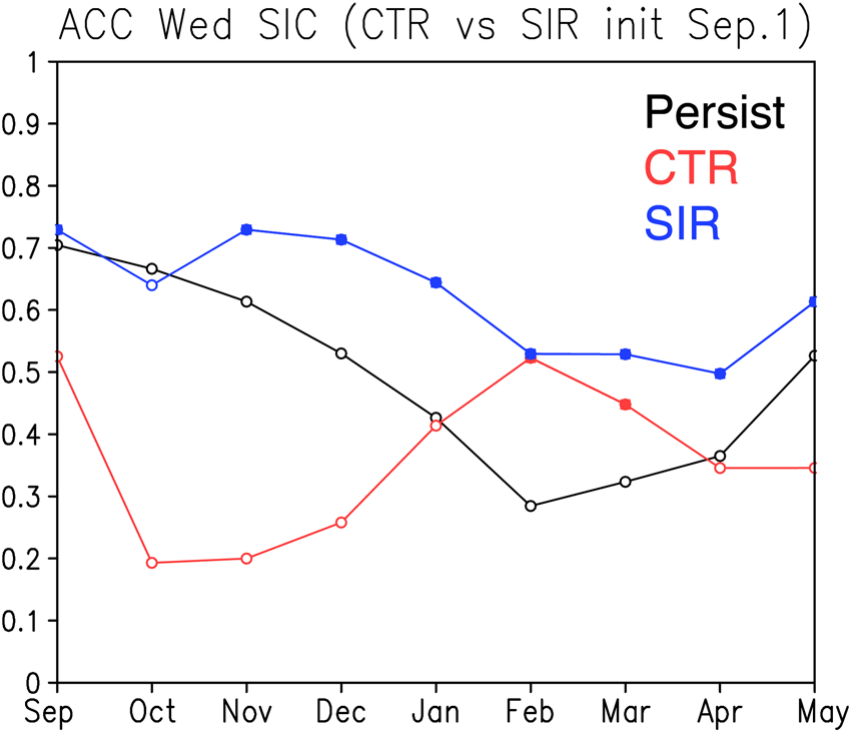
Time series of anomaly correlation coefficients (ACC) of the observed sea-ice concentration (SIC) between August and subsequent months (Persistence; black line). Red (blue) line shows time series of the ACC of the SIC between the observation and the CTR (SIR) reforecast results from September 1st. Solid circles on the CTR and SIR lines indicate the ACCs which exceed the persistence values and are statistically significant at 95% confidence level of a two-tailed Student’s *t*-test. The plots were generated using Grid Analysis and Display System (GrADS) Version 2.1.a3 (http://cola.gmu.edu/grads/downloads.php).

To evaluate the improvement of the SIC prediction skills in the Weddell Sea, the ACCs of the SIC averaged in the Weddell Sea for the two reforecast results from September 1st are plotted against prediction month (Fig. 3). Here we used area-averaged SIC anomalies in the Weddell Sea (60°W-0°, south of 65°S) where the significant improvement of the SIC’s ACC is obtained in Fig. 2d. The persistence value of the observed SIC gradually drops from 0.7 in September to 0.3 in February, while the CTR experiment shows lower ACC than the persistence value until January. On the other hand, the ACC in the SIR experiment exceeds the persistence value over the analysis months except October. In particular, the ACC in the SIR experiment is much higher than that in the CTR experiment from September to January. This indicates that in case of the reforecast results from September 1st, the SIC initialization helps improve the prediction skills of the SIC in the Weddell Sea up to four months ahead.

**Figure 4:**
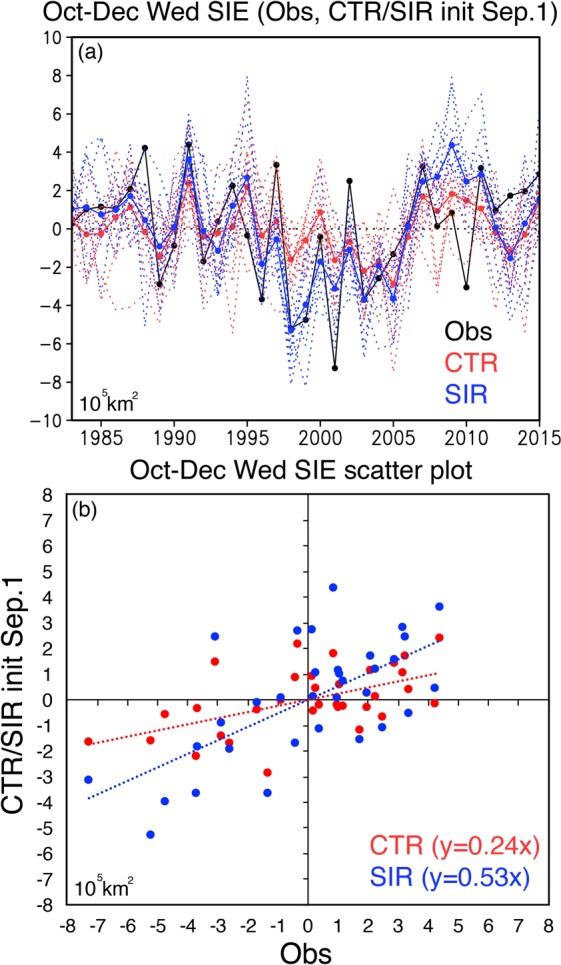
(**a**) Time series of sea-ice extent anomalies (SIE, unit in 10^5^ km^2^) in the Weddell Sea region (60°W-0°) during Oct–Dec of 1983–2015. Black, red, and blue lines correspond to the observations, the CTR and SIR reforecast results from September 1st, respectively. Thick solid red (blue) line denotes the ensemble mean of 12 members represented by thin dashed red (blue) lines. (**b**) Scatter plots of the CTR and SIR reforecast results against the observed SIE anomalies (in 10^5^ km^2^) shown in (**a**). Red and blue dots denote the CTR and SIR reforecast results, while red and blue dashed lines show the regression lines drawn using the least squares method. The plots were generated using Grid Analysis and Display System (GrADS) Version 2.1.a3 (http://cola.gmu.edu/grads/downloads.php) and Microsoft Excel for Mac Version 15.38.

To estimate statistical differences between the CTR and SIR experiments, time series of SIE anomalies in the Weddell Sea (60°W-0°) during Oct–Dec were illustrated in Fig. 4a. It should be noted that sea-ice extent here is defined as the area above 15% of SIC following the conventional definition^4^. The observed SIE anomalies show a pronounced interannual variability with the maximum value (4.39 × 10^5^ km^2^) in 1991 and the minimum value (−7.27 × 10^5^ km^2^) in 2001. While the CTR reforecast results from September 1st tend to underestimate the amplitude of the observed interannual variability, the SIR experiment exhibits a clear improvement with the SIE anomalies that follow the observed ones, for example, the positive peak in 1991 and the negative peaks in the late 1990 s. The predicted SIE anomalies in the Weddell Sea are improved with an increase of the ACC score from 0.54 in the CTR experiment to 0.65 in the SIR experiment and also with a decrease in the root-squared-error (RMS) from 2.39 × 10^5^ km^2^ in the CTR experiment to 2.19 × 10^5^ km^2^ in the SIR experiment, although these differences are not statistically significant. These results suggest that the SIC initialization in the Antarctica improves the predicted amplitude and phase of the SIE anomalies in the Weddell Sea by around 10%. To demonstrate the improvement in a more quantitative way, we plotted the CTR and SIR reforecast results against the observed SIE anomalies in Fig. 4b. The scatter plots represent that the regression line (y = 0.53x) for the SIR reforecast results gets closer to the observation (y = x) by a factor of two compared to the one (y = 0.24x) for the CTR reforecast results. The predicted SIE anomalies in the SIR reforecast results are improved compared to those in the CTR reforecast results in terms of phase and amplitude.

### Composite analysis during low Weddell sea-ice years

Based on the time series of the SIE anomalies, high and low sea-ice years in the Weddell Sea are identified. Following the previous study by Morioka *et al*.^6^, we defined high (low) sea-ice years as ones when the observed SIC anomalies during Nov-Jan exceed one positive (negative) standard deviation. Selection of one-month ahead of Oct–Dec period is because Nov-Jan is one of the two periods when the standard deviation of the SIC anomalies in the Weddell Sea becomes large, although the local atmosphere-ocean-ice interaction takes place more effectively during the Oct–Dec period when the SIC anomalies develop^6^. This procedure leads to identification of five low sea-ice years (1996, 1998, 1999, 2001, and 2010) and seven high sea-ice years (1991, 1994, 2002, 2007, 2011, 2014, and 2015) in the Weddell Sea. Considering the fact that the ACC during Oct–Dec is higher than that during Nov-Jan (Fig. 3), this study focuses on composite sea-ice and climate anomalies during Oct–Dec to identify potential impacts of the SIC initialization.

**Figure 5:**
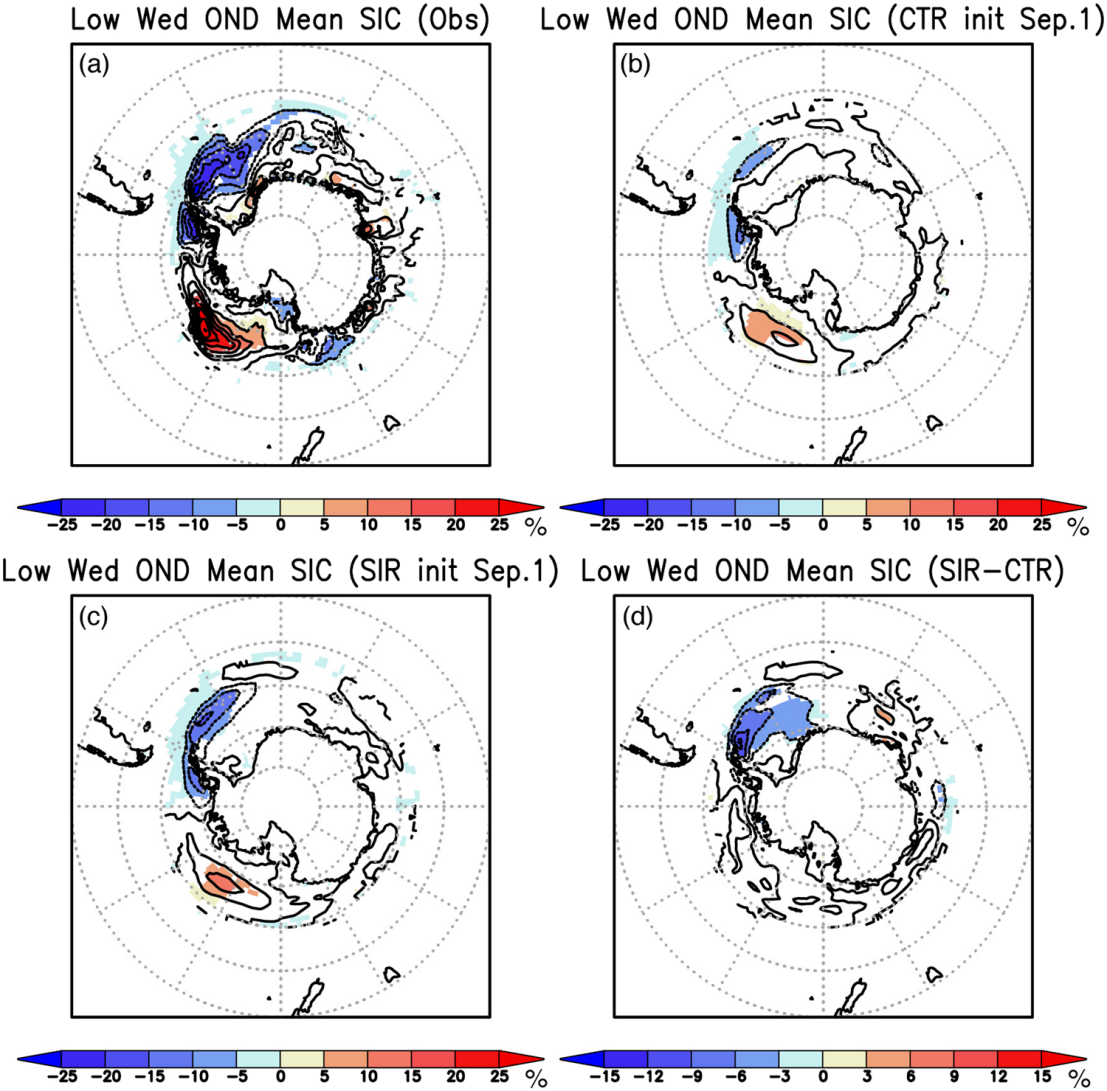
(**a**) Composite anomalies of the observed sea-ice concentration (SIC, color and contour in %) during Oct–Dec of lower-than-normal Weddell sea-ice years (1996, 1998, 1999, 2001, and 2010). Anomalies statistically significant at 95% confidence level using a two-tailed Student’s *t*-test are shaded. (**b**) Same as in (**a**), but for the CTR reforecast results from September 1st. (**c**) Same as in (**b**), but for the SIR reforecast results from September 1st. (**d**) Same as in (**b**), but for differences in the anomalies between the SIR and CTR reforecast results. Anomaly differences statistically significant at 95% confidence level using a two-tailed Student’s *t*-test are shaded. The maps were generated using Grid Analysis and Display System (GrADS) Version 2.1.a3 (http://cola.gmu.edu/grads/downloads.php).

Multiple comparison of SIC anomalies among the observation and reforecast results is made in Fig. 5. During Oct–Dec season of the low Weddell sea-ice years, negative SIC anomalies are strongly accompanied by the positive SIC anomalies in the Ross Sea (Fig. 5a). The spatial pattern of the SIC anomalies is well predicted in both the CTR and SIR reforecast results from September 1st (Fig. 5b,c), but the amplitude of negative SIC anomalies in the Weddell Sea is more accurately captured in the SIR experiment, while in the Ross Sea, the positive SIC anomalies show a similar amplitude. This can be clearly seen in the statistically significant differences in the negative SIC anomalies over the Weddell Sea between the SIR and CTR experiments (Fig. 5d).

The comparison between the SIR and CTR experiments indicates that the negative SIC anomalies during the low Weddell sea-ice years can be predicted to some extent even with the SST initialization only. But in the presence of the SIC initialization, the amplitude of the SIC anomalies in the Weddell Sea is further improved. These results are consistent with the fact that the ACC in the SIR experiment during Oct–Dec is higher than that in the CTR experiment (Fig. 3). The local atmosphere-ocean-ice interaction may play more roles than remote forcing in generating the negative SIC anomalies of the Weddell Sea, while in the Ross Sea, due to proximity to the tropical Pacific, the positive SIC anomalies may be more induced by atmospheric teleconnection from the tropical Pacific. This argument is also consistent with previous results based on the observational analysis^19^.

**Figure 6:**
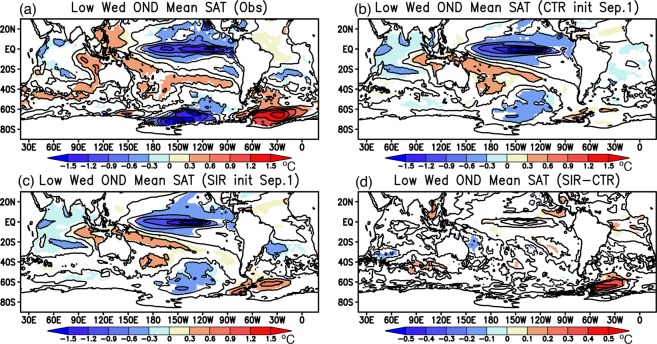
Same as in Fig. 5, but for the surface air temperature anomalies (SAT, color and contour in °C). For convenience of interpretation, anomalies over the continent are masked out. The maps were generated using Grid Analysis and Display System (GrADS) Version 2.1.a3 (http://cola.gmu.edu/grads/downloads.php).

To obtain some useful insights into the influence of the SIC initialization on the atmospheric variability, further comparison is made in terms of SAT anomalies (Fig. 6). During Oct–Dec of low Weddell sea-ice years, the high SAT anomalies are observed over the Weddell Sea, suggesting more incoming shortwave radiation to increase the SAT due to the decreased surface albedo (Fig. 6a). The positive SAT anomalies are accompanied with the negative SAT anomalies over the Ross Sea where the high SIC anomalies are observed (Fig. 5a). It is worth mentioning that these SAT anomalies are strongly associated with the negative SAT anomalies over the tropical Pacific. In fact, three out of five low Weddell sea-ice years are accompanied with La Niña years (1998, 1999, and 2010). The association between the positive SAT anomalies over the Weddell Sea and the La Niña events is well predicted in the CTR and SIR experiments (Fig. 6b,c). However, differences in the SAT anomalies between the SIR and CTR experiments represent the statistically significant improvement of the SAT anomalies mainly over the Weddell Sea region (Fig. 6d). There are some regions outside the Weddell Sea with weak differences in the SAT anomalies between the CTR and SIR experiments, but the differences are very small and not much significant, compared to those in the positive SAT anomalies in the Weddell Sea (around 0.5 °C).

**Figure 7:**
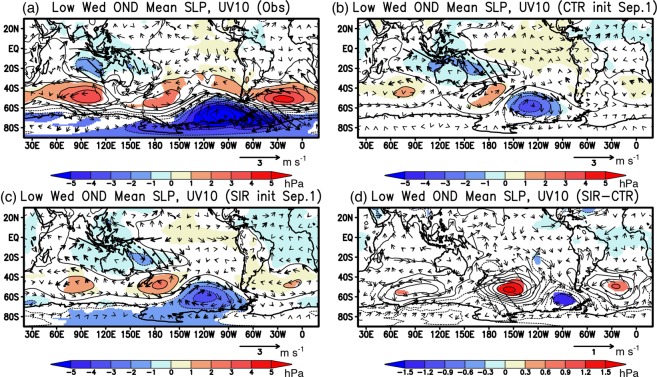
Same as in Fig. 5, but for the sea-level pressure anomalies (SLP; color and contour in hPa) and the horizontal wind anomalies at 10 m (UV10; arrow in m s^−1^) above the surface. Thick arrows indicate wind anomalies statistically significant at 95% confidence level of a two-tailed Student’s *t*-test. The maps were generated using Grid Analysis and Display System (GrADS) Version 2.1.a3 (http://cola.gmu.edu/grads/downloads.php).

To illustrate the impact of sea-ice initialization on the atmospheric circulation anomalies, composite sea-level pressure (SLP) anomalies during Oct–Dec of low Weddell sea-ice years were calculated in Fig. 7. As reported in a previous study^6^, the low Weddell sea-ice anomalies are associated with high SLP anomalies to the northeast and low SLP anomalies to the southwest (Fig. 7a). In particular, the low SLP anomalies west of the Antarctic Peninsula are accompanied with the high SLP anomalies southeast of New Zealand, indicating remote influence of the atmospheric teleconnection from the tropical Pacific. On the other hand, the meridional seesaw pattern of the low SLP anomalies over the Antarctica and the high SLP anomalies in the mid latitudes is well-pronounced, suggesting another atmospheric forcing from a positive phase of the SAM.

However, there is no denying that the low Weddell sea-ice anomalies may help sustain high SLP anomalies in the South Atlantic through a decrease in the near-surface baroclinicity due to a decrease in the meridional temperature gradient^6^. Both CTR and SIR reforecast results from September 1st show high SLP anomalies with anticyclonic surface wind anomalies in the Atlantic sector of the Southern Ocean and atmospheric teleconnection from the tropical Pacific (Fig. 7b,c), but the SIR experiment better predicts higher SLP anomalies in the Atlantic sector and positive sign of the SAM in the mid-high latitudes (Fig. 7c). The differences in the SLP anomalies between the SIR and CTR experiments exhibit statistically significant differences in the high SLP anomalies over the Weddell Sea, although the differences in the anticyclonic surface wind anomalies are not statistically significant (Fig. 7d). Since there are weak differences in the SAT anomalies outside of the Weddell Sea region between the CTR and SIR experiments, the high SAT anomalies in the Weddell Sea (Fig. 6d) may help improve prediction of the high SLP anomalies in the Weddell Sea through local atmosphere-ocean-ice interaction.

**Figure 8:**
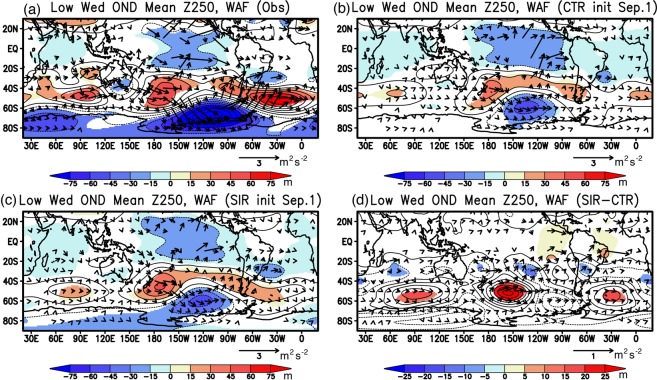
Same as in Fig. 5, but for the geopotential height anomalies (Z250; color and contour in hPa) and the wave activity fluxes (WAF; arrow in m^2^ s^−2^) at 250 hPa. The maps were generated using Grid Analysis and Display System (GrADS) Version 2.1.a3 (http://cola.gmu.edu/grads/downloads.php).

To further investigate the downstream influence of the atmospheric response to the SIC anomalies over the Weddell Sea, we calculated the geopotential height anomalies and wave activity fluxes^20,21^ at 250 hPa that portray the group velocity and propagation pathways of stationary Rossby waves (Fig. 8). The atmospheric reanalysis results exhibit clear wave trains of positive and negative geopotential height anomalies in the Southern Ocean (Fig. 8a). On the other hand, the eastward propagation of the stationary Rossby waves to the positive geopotential height anomalies in the Atlantic sector are predicted much weaker in both the CTR and SIR experiments (Fig. 8b,c). It is worth noting that the differences in geopotential height anomalies between the two experiments show anticyclonic circulation anomalies to the north of the Weddell Sea (Fig. 8d), indicating an equivalent barotropic structure in the whole troposphere. The anomalies also emit the stationary Rossby waves downstream to the negative and positive geopotential height anomalies in the Indian Ocean sector of the Southern Ocean (Fig. 8d). The analysis of the atmospheric variability in the upper troposphere suggests that improvement of anticyclonic circulation anomalies to the north of the Weddell Sea may be more related to that of local atmosphere-ocean-ice interaction involving the SIC anomalies in the Weddell Sea.

### Composite analysis during high Weddell sea-ice years

Improvement for prediction of the SIC and atmospheric variability is found very limited during the high Weddell sea-ice years. Composite SIC anomalies observed during Oct–Dec season show anomalously high SIC anomalies in the Weddell Sea (Fig. S1a), but the high SIC anomalies in both the CTR and SIR experiments are much weaker than the observed SIC anomalies (Fig. S1b,c). The differences in the SIC anomalies between the two experiments reveal statistically significant positive SIC anomalies in the Weddell Sea (Fig. S1d), though the amplitude is weaker than that in the case of the negative SIC anomalies during the low Weddell sea-ice years (Fig. 5d). Because of this weaker amplitude of the positive SIC anomalies, the negative SAT anomalies observed over the Weddell Sea during Oct–Dec (Fig. S2a) are weakly predicted in both the CTR and SIR experiments (Fig. S2b,c). Also, the differences in the SAT anomalies between the two experiments are weakly negative but not statistically significant (Fig. S2d).

A similar tendency is obtained for the atmospheric circulation variability. Composite SLP anomalies during Oct–Dec exhibit cyclonic circulation anomalies to the north of the Weddell Sea (Fig. S3a). However, both the CTR and SIR experiments do not reproduce the negative SLP anomalies well, although they reasonably capture the positive SLP anomalies west of the Antarctica (Fig. S3b,c). These results suggest a weak atmospheric teleconnection between the South Pacific and the Weddell Sea in these experiments. The differences in the SLP anomalies between the two experiments are also not statistically significant to the north of the Weddell Sea (Fig. S3d). In the upper troposphere, there are clear wave trains of negative and positive geopotential height anomalies observed over the South Pacific (Fig. S4a), which are well captured in the CTR and SIR experiments (Fig. S4b,c). However, neither experiments could predict negative geopotential height anomalies to the north of the Weddell Sea. Also, no significant improvement is obtained over the Weddell Sea in the SIR experiment (Fig. S4d). The weaker reproduction of the atmospheric variability over the Weddell Sea during the high sea-ice years may be attributed to those of the SIC anomalies in the Weddell Sea that act to reduce the local atmosphere-ocean-ice interaction (Fig. S1b–d).

## Discussions

Present study has identified a potential influence of the Antarctic SIC initialization on seasonal prediction of the regional atmospheric variability in the Weddell Sea and the Atlantic sector of the Southern Ocean. Previous studies^9–12^ have reported that the atmospheric variability in the region is influenced by atmospheric teleconnection from the tropical Pacific. But, a recent study has suggested a possible influence of local sea-ice variation on the atmospheric variability through changes in the near-surface baroclinicity^6^. Comparison between the two reforecast experiments with and without the SIC initialization reveals that a reasonable representation of sea-ice condition is necessary for better simulation and prediction of the atmospheric variability associated with the sea-ice variability. In particular, during the low sea-ice years, the predicted SIC anomalies over the Weddell Sea tend to locally modulate the atmospheric variability through atmosphere-ocean-ice interaction, because almost no significant differences in the predicted SAT anomaly patterns between the two experiments are found outside the Weddell Sea (Fig. 6b), for example in the tropical Pacific, probably due to the effect of SST initialization^22^.

The extent of realistic sea-ice variability through the model initialization is further evaluated by analyzing composite SIC anomalies during austral winter of the low Weddell sea-ice years (Fig. S5). Three-month average of initialized results over Jun-Aug was performed here to be consistent with the previous discussion on the seasonally mean reforecast results from September 1st. The observations clearly show a decrease in the SIC near the Antarctic Peninsula and an increase in the SIC in the Ross Sea, respectively (Fig. S5a). The spatial patterns of the observed SIC anomalies are well simulated in the CTR and SIR experiments (Fig. S5b,c), but the negative SIC anomalies in the Weddell Sea are better represented in the presence of the SIC initialization (Fig. S5d). The improvement of initial SIC anomalies also leads to that of the initial SAT anomalies in the model experiments (Fig. S6). The high SAT anomalies observed over the Weddell Sea (Fig. S6a) are simulated in the CTR and SIR experiments (Fig. S6b,c). The differences between the two experiments indicate significant improvement of the SAT anomalies over the Weddell Sea in the SIR experiment (Fig. S6d). This may be due to better representation of low SIC anomalies in the region (Fig. S5d).

However, the improvement of initial SIC anomalies in the Weddell Sea does not lead to better representation of initial SLP anomalies there (Fig. S7). During Jun-Aug, the atmospheric reanalysis results show the pronounced negative SLP anomalies over the Weddell Sea (Fig. S7a), while the CTR experiment simulates positive SLP anomalies there (Fig. S7b). The positive SLP anomalies over the Weddell Sea become weaker in the SIR experiment (Fig. S7c), although the differences in the SLP anomalies between the SIR and CTR experiments are not statistically significant with negative values (Fig. S7d). The analysis of initial SIC and atmospheric conditions during Jun-Aug suggests that improvement of the predicted SLP anomalies during Oct–Dec may not be directly linked to better representation of initial atmospheric circulation anomalies, but due to persistence of the initial SIC anomalies from austral winter to austral spring and the associated atmosphere-ocean-ice interaction.

Furthermore, the improvement of the prediction skills in the Weddell Sea region is found to depend on the initialization month of the reforecast experiment (Fig. S8). For example, in case of the reforecast results from July 1st, the ACC in the SIR experiment exceeds that in the CTR experiment in the first four months up to October (Fig. S8a), whereas in the case of the reforecast results from May 1st, the ACC in the SIR experiment is not so different from that of the CTR experiment (Fig. S8b). Also, the ACCs in both experiments are lower than the persistence skills, indicating difficulty in predicting the SIC anomalies across austral winter (Jun-Aug). The different behavior of the ACCs suggests that the SIC initialization during austral winter when the sea-ice cover approaches to its maximum may provide more favorable conditions for the SIC prediction in austral spring.

Considering the persistence of the SIC anomalies from austral winter to spring, persistence forecast might be useful for predicting the atmospheric variability associated with the sea-ice anomalies. Indeed, the persistence forecast for the SAT anomalies shows moderately high ACC over the Weddell Sea (Fig. S9a), but both the CTR and SIR experiments add further positive values to the persistence forecast skills in the western part of the Weddell Sea (Fig. S9b–d). On the other hand, the persistence forecast for the SLP anomalies shows very limited skills in the mid- and high-latitudes of the Southern Hemisphere (Fig. S10a), in contrast to those in the CTR and SIR experiments (Fig. S10b,c). The prediction skills for the SLP anomalies become better to the north of the Weddell Sea in the SIR experiments (Fig. S10d). Therefore, prediction skills in the CTR and SIR experiments are more reliable than that of the persistence forecast.

Along this line, accurate initialization of sea ice conditions is imperative for better simulation and prediction in the atmospheric variability of the mid-high latitudes. Since the SIC variability is sensitive to the sea-ice thickness (SIT) condition, initializing the SIT with long-term ocean-ice reanalysis^17^ or relatively short satellite observations^23^ would further improve prediction of SIC variability and hence the overlying atmospheric circulation variability. On the other hand, subsurface ocean conditions such as temperature and salinity have great influences on the SIC and SIT through changes in the ocean temperature and circulation below the sea ice. In this regard, combining SIC/SIT and subsurface ocean initialization using a CGCM would provide better understanding and precise prediction of the sea-ice variability in the Weddell Sea and the associated atmospheric variability in the Atlantic sector of the Southern Ocean.

## Methodology

To evaluate prediction accuracy of CGCM, we utilized the monthly mean SST and SIC data during 1982–2016 from the Optimum Interpolation SST (OISST) v2^24^, which has a 1° × 1° horizontal resolution. Also, we employed the monthly mean SLP, horizontal winds at 10 m, and geopotential height at 250 hPa from the ERA-Interim atmospheric reanalysis, which has the same horizontal resolution^25^. To calculate monthly anomalies, we subtracted monthly climatology and removed a linear trend using a least squares method.

Reforecast experiments were performed using a CGCM developed under Japan-EU collaboration, called the Scale Interaction Experiment-Frontier Research Center for Global Change 2 (SINTEX-F2) model^26,27^. The SINTEX-F2 model is an upgraded version of SINTEX-F1 model^28,29^. The atmospheric component is based on ECHAM5^30^, which has 31 levels in the vertical on a T106 Gaussian grid. The oceanic component of the SINTEX-F2 is Nucleus for European Modeling of the Ocean 3 (NEMO3)^31^, which includes the Louvain-la-Neuve Sea Ice Model 2 (LIM2) sea ice model^32^ and has 0.5° × 0.5° horizontal resolution of ORCA configuration (ORCA05) with 31 levels in the vertical. Both the atmospheric and oceanic fields are exchanged every 2 hours with no flux correction by means of the Ocean Atmosphere Sea Ice Soil 3 (OASIS3) coupler^33^.

To examine potential impacts of sea-ice initialization on the climate predictability, we conducted two reforecast experiments: one is employed as control (CTR) experiment in which the model’s SST is initialized with the observed SST through adjustment of surface heat flux every month from 1982 afterwards^22^. The other is sea-ice restoring (SIR) experiment in which the model’s SIC is additionally restored to the observed SIC over both the Antarctic and Arctic regions. After 32-yr spun-up with the observed SST climatology since 1950, the model was initialized every month from 1982 to 2015 with two initialization schemes. Then, reforecast experiments were performed from 1st date of every month from 1982 to 2015, and the model was freely integrated over 9-month period.

To estimate the model’s uncertainty related to the initial values, we prepared 12 different initial conditions: we first employed two different SST datasets of the weekly OISST v2^24^ and the high-resolution daily NOAA OISST analysis^34^. Then, we adopted three negative feedback values (−2400, −1200, and −800 W m^−2^ K^−1^) to the surface heat flux in order to restore the model’s SST to the observed SST, which corresponds to 1, 2, and 3-day relaxation time for the 50-m mixed-layer temperature, respectively. Finally, we integrated the model with and without the vertical mixing scheme introduced by Sasaki *et al*.^35^. In the scheme, the model takes into account the strong vertical mixing associated with small vertical scale structures (SVSs) within and above the equatorial thermocline. These different initial conditions generate 12 ensemble members for the CTR and SIR experiments. For the SIC initialization, we restored the model’s SIC to the observed SIC with a timescale of 5 days. For each member, we removed monthly climatology over lead months which includes the model drift (systematic error) defined as differences in monthly climatology between the model and the historical datasets. Then, we removed a linear trend over the 33-yr integration period for each lead time to obtain monthly anomalies.

## Supplementary information

Supplementary Figures file
