# Supplementary material for: Role of sea-ice initialization in climate predictability over the Weddell Sea
Source: Sci Rep. 2019 Feb 25;9:2457. doi: 10.1038/s41598-019-39421-w (PMC6387978; doi:10.1038/s41598-019-39421-w)
Supplement: Supplementary file 1 — Supplementary Figures file [file 41598_2019_39421_MOESM1_ESM.pdf]

Supplementary information
"Role of sea-ice initialization in climate predictability over the Weddell Sea"

Yushi Morioka[1], Takeshi Doi[1], Doroteaciro Iovino[2],
Simona Masina[2], Swadhin K. Behera[1]

1: Application Laboratory, JAMSTEC, Yokohama, Japan
2: Fondazione Centro Euro-Mediterraneo sui Cambiamenti Climatici (CMCC), Bologna, Italy
Corresponding author: Dr. Yushi Morioka
E-mail: morioka@jamstec.go.jp

Supplementary Figure Captions

Figure S1: (a) Composite anomalies of the observed sea-ice concentration (SIC, color and contour in %) during Oct-Dec of higher-than-normal Weddell sea-ice years (1991, 1994, 2002, 2007, 2011, 2014, and 2015). Anomalies statistically significant at 95% confidence level using a two-tailed Student's *t*-test are shaded. (b) Same as in (a), but for the CTR reforecast results from September 1st. (c) Same as in (b), but for the SIR reforecast results from September 1st. (d) Same as in (b), but for differences in the anomalies between the SIR and CTR reforecast results. Anomaly differences statistically significant at 95% confidence level using a two-tailed Student's *t*-test are colored. The maps were generated using Grid Analysis and Display System (GrADS) Version 2.1.a3 (http://cola.gmu.edu/grads/downloads.php).

Figure S2: Same as in Fig. S1, but for the surface air temperature anomalies (SAT, color and contour in ºC). For convenience of interpretation, anomalies over the continent are masked out. The maps were generated using Grid Analysis and Display System (GrADS) Version 2.1.a3 (http://cola.gmu.edu/grads/downloads.php).

Figure S3: Same as in Fig. S1, but for the sea-level pressure anomalies (SLP; color and contour in hPa) and the horizontal wind anomalies at 10 m (UV10; arrow in $m\ s^{-1}$) above the surface. Thick arrows indicate the wind anomalies statistically significant at 95% confidence level of a two-tailed Student's *t*-test. The maps were generated using Grid Analysis and Display System (GrADS) Version 2.1.a3 (http://cola.gmu.edu/grads/downloads.php).

Figure S4: Same as in Fig. S1, but for the geopotential height anomalies (Z250; color and contour in hPa) and the wave activity fluxes (WAF; arrow in $m^2\ s^{-2}$) at 250 hPa. The maps were generated using Grid Analysis and Display System (GrADS) Version 2.1.a3 (http://cola.gmu.edu/grads/downloads.php).

Figure S5: (a) Composite anomalies of the observed sea-ice concentration (SIC, color and contour in %) during Jun-Aug of low Weddell sea-ice years. Anomalies statistically significant at 95% confidence level using a two-tailed Student's *t*-test are shaded. (b) Same as in (a), but for the CTR reforecast results during the initialization phase (Jun-Aug) of lower-than-normal Weddell sea-ice years. (c) Same as in (b), but for the SIR reforecast results. (d) Same as in (b), but for differences in the anomalies between the SIR and CTR reforecast results. Anomaly differences statistically significant at 95% confidence level using a two-tailed Student's *t*-test are shaded. The maps were generated using Grid Analysis and Display System (GrADS) Version 2.1.a3 (http://cola.gmu.edu/grads/downloads.php).

Figure S6: Same as in Fig. S5, but for the surface air temperature anomalies (SAT, color and contour in ºC). For convenience of interpretation, anomalies over the continent are masked out. The maps were generated using Grid Analysis and Display System (GrADS) Version 2.1.a3 (http://cola.gmu.edu/grads/downloads.php).

Figure S7: Same as in Fig. S5, but for the sea level pressure anomalies (SLP, color and contour in hPa) and the horizontal wind anomalies at 10 m (UV10; arrow in $m\ s^{-1}$) above the surface. Thick arrows indicate the wind anomalies statistically significant at 95% confidence level of a two-tailed Student's *t*-test. The maps were generated using Grid Analysis and Display System (GrADS) Version 2.1.a3 (http://cola.gmu.edu/grads/downloads.php).

Figure S8: (a) Time series of anomaly correlation coefficients (ACC) of the observed sea-ice concentration (SIC) between June and subsequent months (Persistence; black line). Red (blue) line shows time series of the ACC between the observation and the CTR (SIR) reforecast results from July 1st. Solid circles on the CTR and SIR lines indicate the ACCs which exceed the persistence values and are statistically significant at 95% confidence level of a two-tailed Student's *t*-test. (b) Same as in (a), but for the case of the CTR (SIR) reforecast results from May 1st. The maps were generated using Grid Analysis and Display System (GrADS) Version 2.1.a3 (http://cola.gmu.edu/grads/downloads.php).

Figure S9: (a) Anomaly correlation coefficient (ACC; Persistence) of the observed surface air temperature (SAT) anomalies between Jun-Aug and Oct-Dec seasons. The statistically significant ACCs at 95% confidence level of a two-tailed Student's *t*-test are shaded. (b) ACC of the Oct-Dec mean SAT anomalies between the observation and the CTR reforecast results from September 1st. Positive ACCs which exceed persistence values in (a) and are statistically significant at 95% confidence level of a two-tailed Student's *t*-test are shaded. (c) Same as in (b), but from the SIR reforecast results from September 1st. (d) Differences in the ACCs between the SIR and CTR reforecast results. The maps were generated using Grid Analysis and Display System (GrADS) Version 2.1.a3 (http://cola.gmu.edu/grads/downloads.php).

Figure S10: Same as in Fig. S9, but for the sea-level pressure (SLP) anomalies. The maps were generated using Grid Analysis and Display System (GrADS) Version 2.1.a3 (http://cola.gmu.edu/grads/downloads.php).

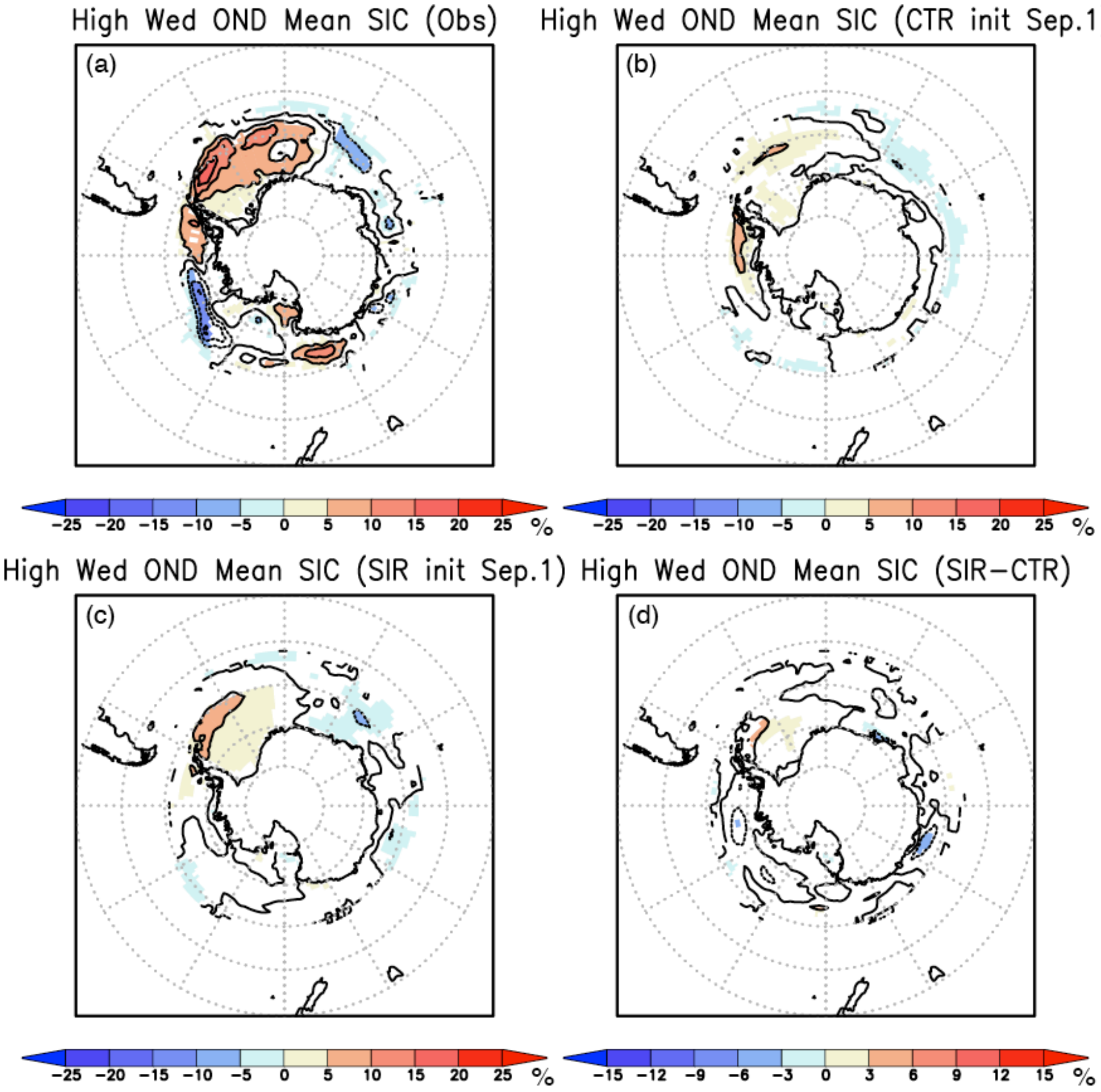

Figure S1: (a) Composite anomalies of the observed sea-ice concentration (SIC, color and contour in %) during Oct-Dec of higher-than-normal Weddell sea-ice years (1991, 1994, 2002, 2007, 2011, 2014, and 2015). Anomalies statistically significant at 95% confidence level using a two-tailed Student's *t*-test are shaded. (b) Same as in (a), but for the CTR reforecast results from September 1st. (c) Same as in (b), but for the SIR reforecast results from September 1st. (d) Same as in (b), but for differences in the anomalies between the SIR and CTR reforecast results. Anomaly differences statistically significant at 95% confidence level using a two-tailed Student's *t*-test are colored. The maps were generated using Grid Analysis and Display System (GrADS) Version 2.1.a3 (http://cola.gmu.edu/grads/downloads.php).

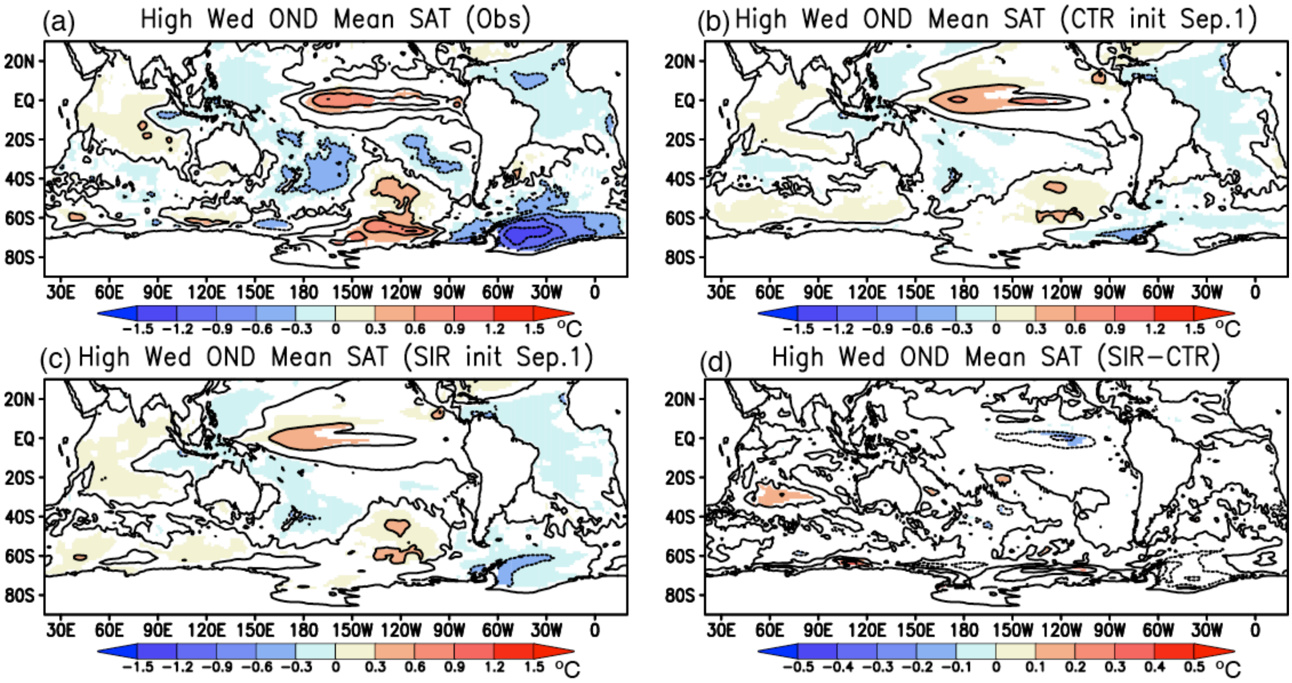

Figure S2: Same as in Fig. S1, but for the surface air temperature anomalies (SAT, color and contour in ºC). For convenience of interpretation, anomalies over the continent are masked out. The maps were generated using Grid Analysis and Display System (GrADS) Version 2.1.a3 (http://cola.gmu.edu/grads/downloads.php).

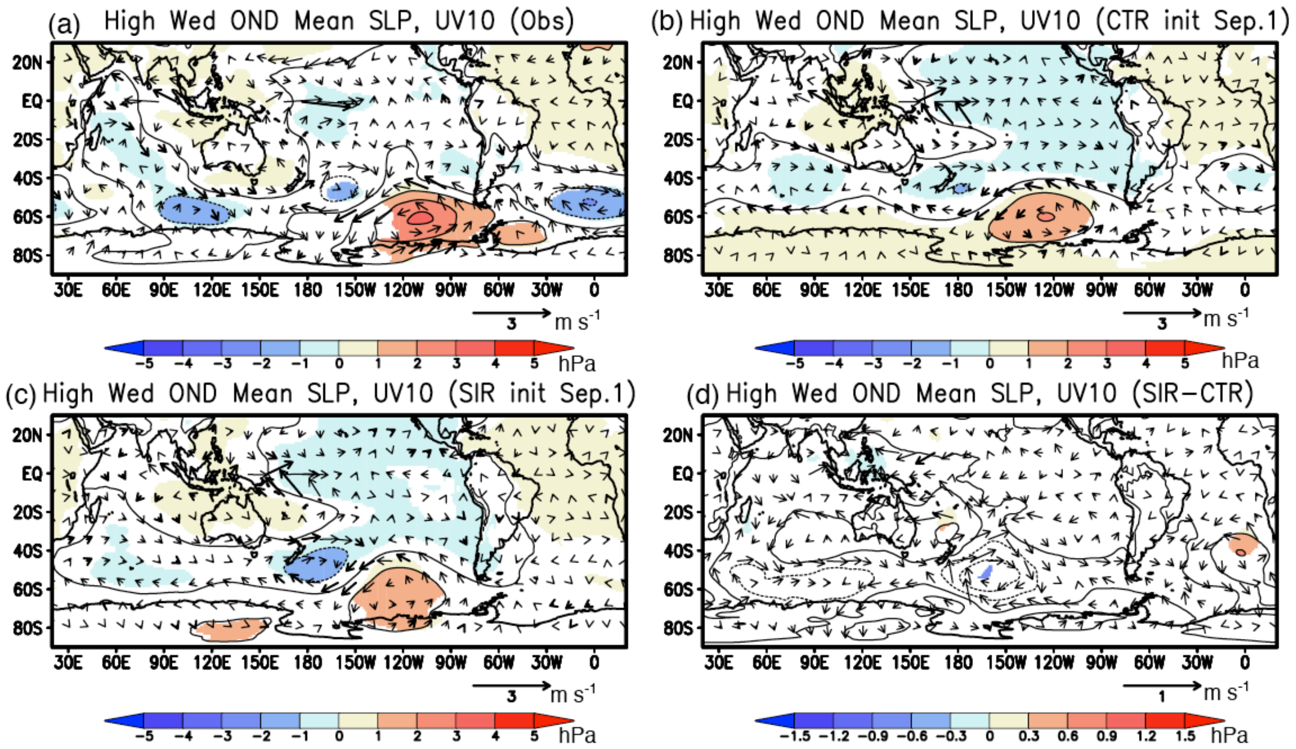

Figure S3: Same as in Fig. S1, but for the sea-level pressure anomalies (SLP; color and contour in hPa) and the horizontal wind anomalies at 10 m (UV10; arrow in m s^{-1}) above the surface. Thick arrows indicate the wind anomalies statistically significant at 95% confidence level of a two-tailed Student's *t*-test. The maps were generated using Grid Analysis and Display System (GrADS) Version 2.1.a3 (http://cola.gmu.edu/grads/downloads.php).

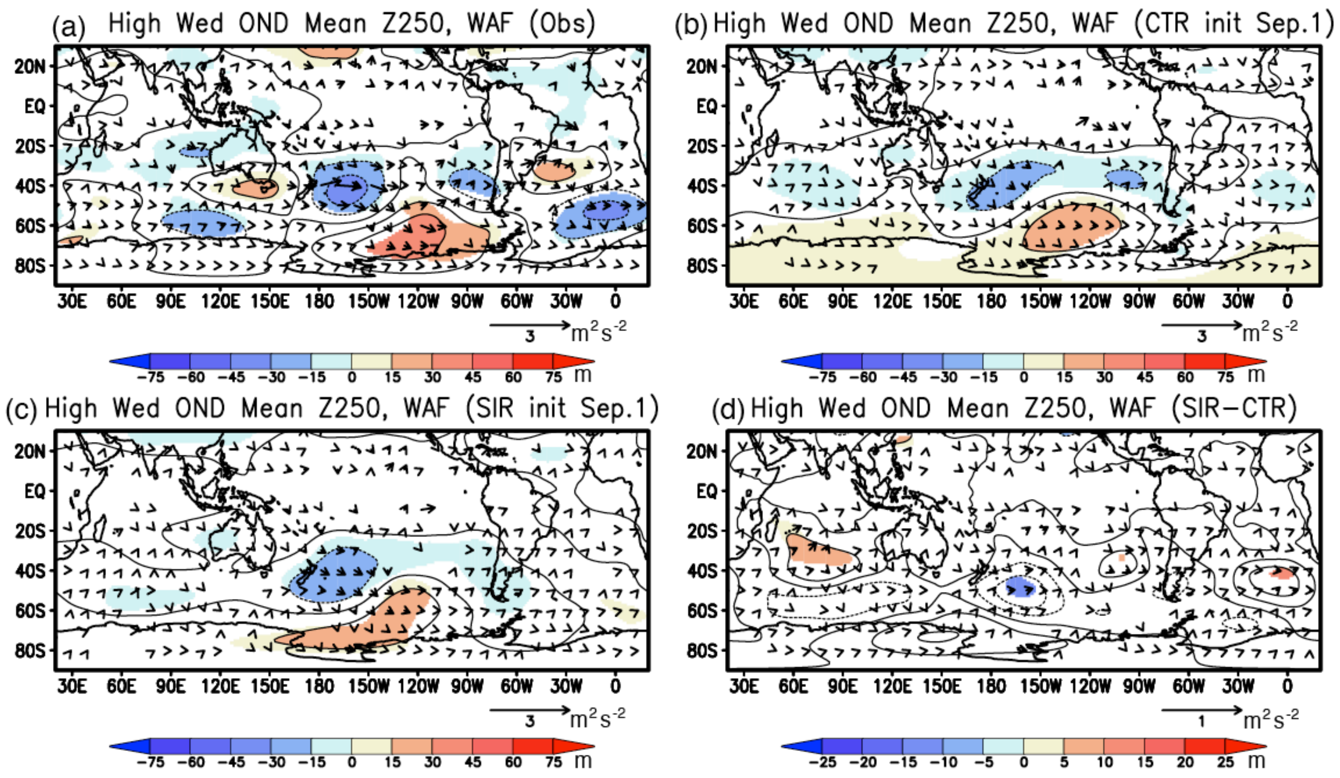

Figure S4: Same as in Fig. S1, but for the geopotential height anomalies (Z250; color and contour in hPa) and the wave activity fluxes (WAF; arrow in $m^2 s^{-2}$) at 250 hPa. The maps were generated using Grid Analysis and Display System (GrADS) Version 2.1.a3 (http://cola.gmu.edu/grads/downloads.php).

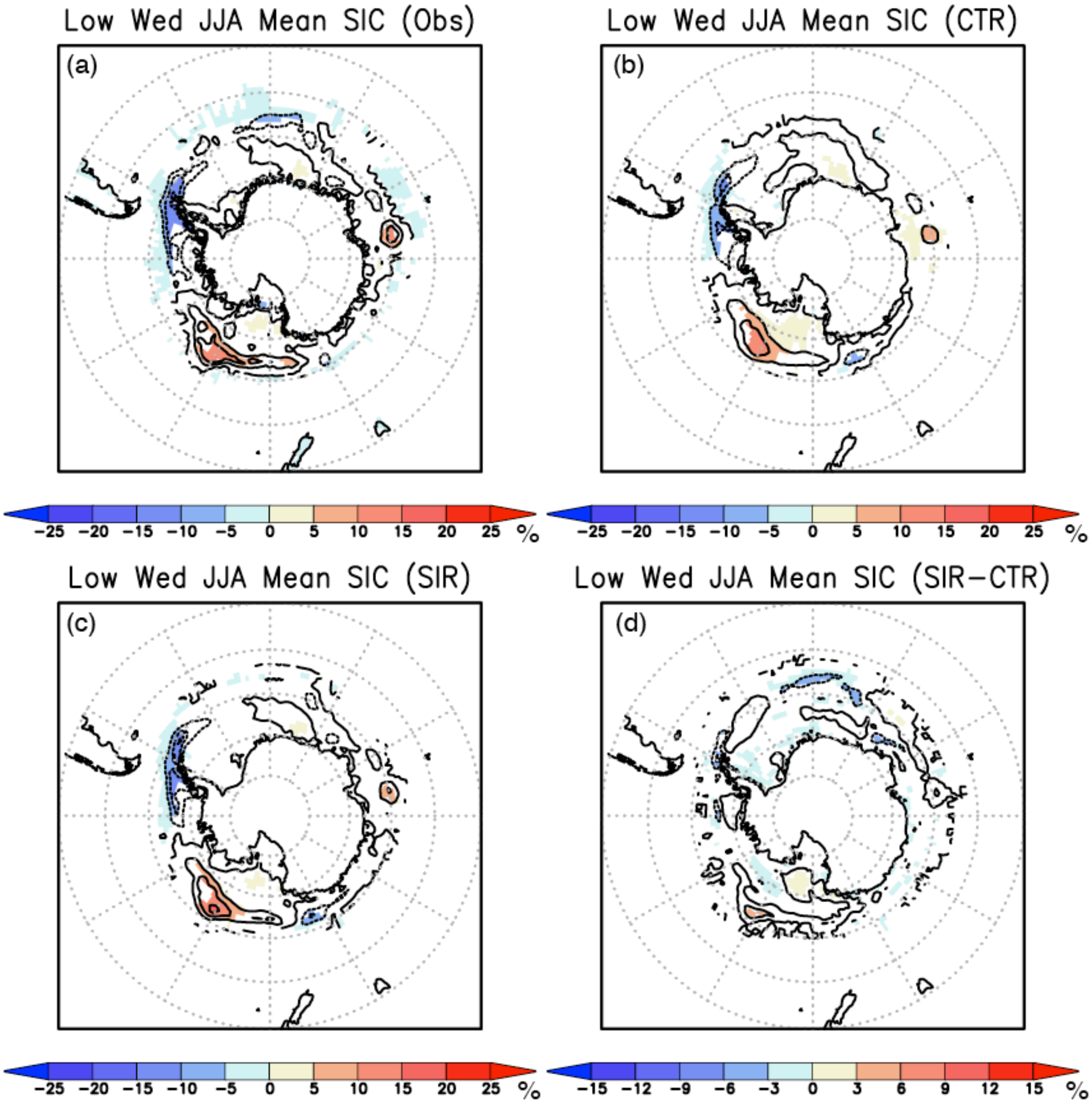

Figure S5: (a) Composite anomalies of the observed sea-ice concentration (SIC, color and contour in %) during Jun-Aug of low Weddell sea-ice years. Anomalies statistically significant at 95% confidence level using a two-tailed Student's *t*-test are shaded. (b) Same as in (a), but for the CTR reforecast results during the initialization phase (Jun-Aug) of lower-than-normal Weddell sea-ice years. (c) Same as in (b), but for the SIR reforecast results. (d) Same as in (b), but for differences in the anomalies between the SIR and CTR reforecast results. Anomaly differences statistically significant at 95% confidence level using a two-tailed Student's *t*-test are shaded. The maps were generated using Grid Analysis and Display System (GrADS) Version 2.1.a3 (http://cola.gmu.edu/grads/downloads.php).

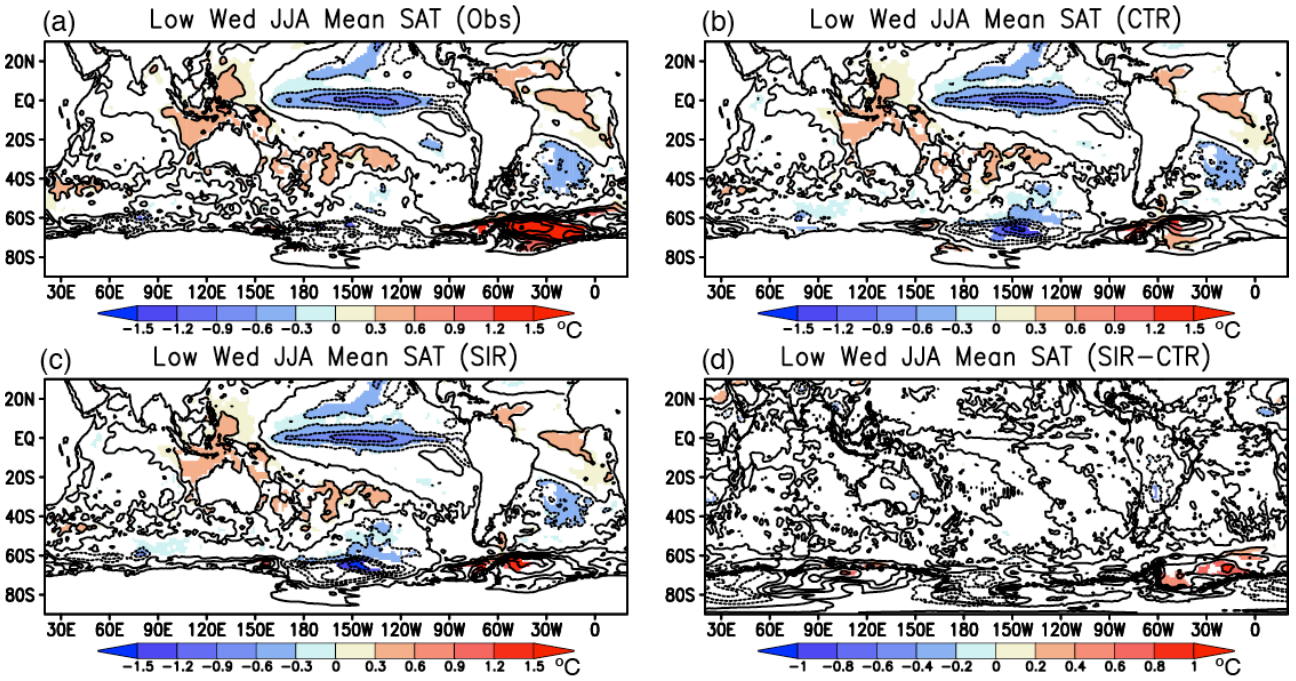

Figure S6: Same as in Fig. S5, but for the surface air temperature anomalies (SAT, color and contour in ºC). For convenience of interpretation, anomalies over the continent are masked out. The maps were generated using Grid Analysis and Display System (GrADS) Version 2.1.a3 (http://cola.gmu.edu/grads/downloads.php).

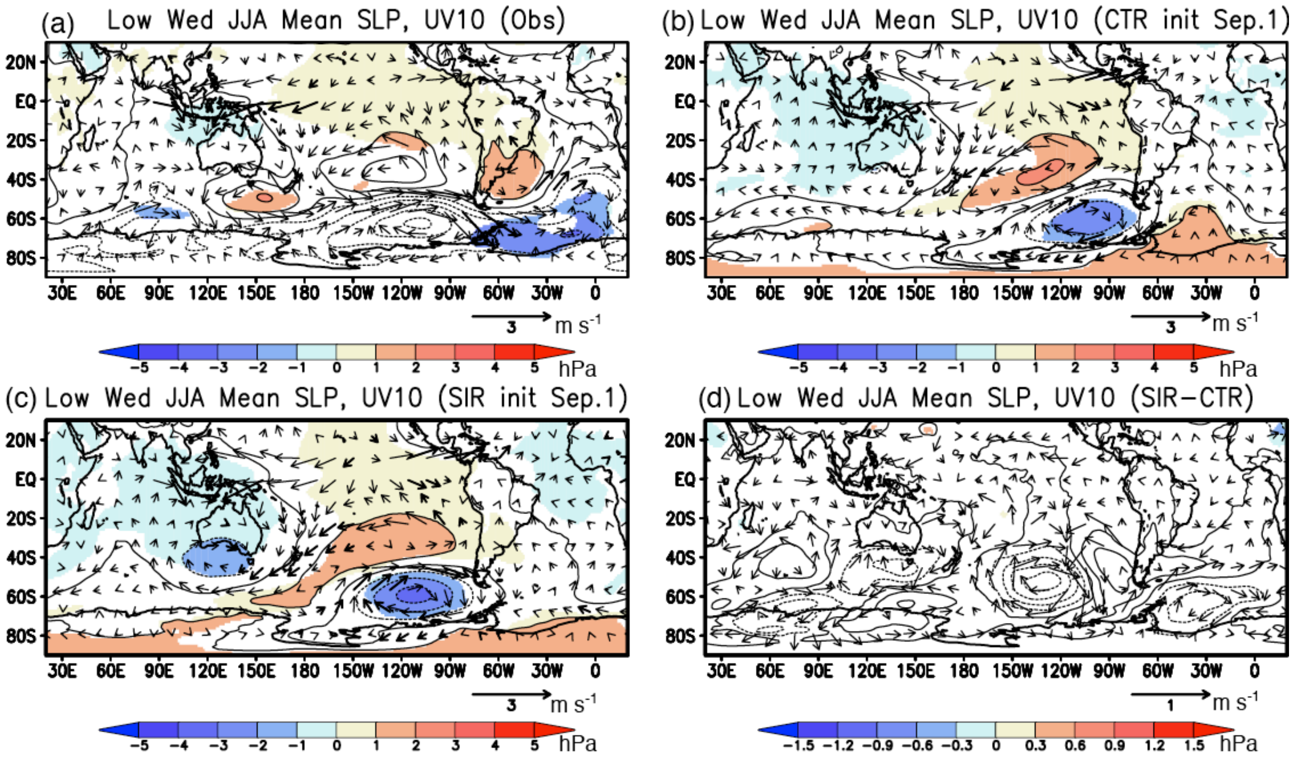

Figure S7: Same as in Fig. S5, but for the sea level pressure anomalies (SLP, color and contour in hPa) and the horizontal wind anomalies at 10 m (UV10; arrow in m s^{-1}) above the surface. Thick arrows indicate the wind anomalies statistically significant at 95% confidence level of a two-tailed Student's *t*-test. The maps were generated using Grid Analysis and Display System (GrADS) Version 2.1.a3 (http://cola.gmu.edu/grads/downloads.php).

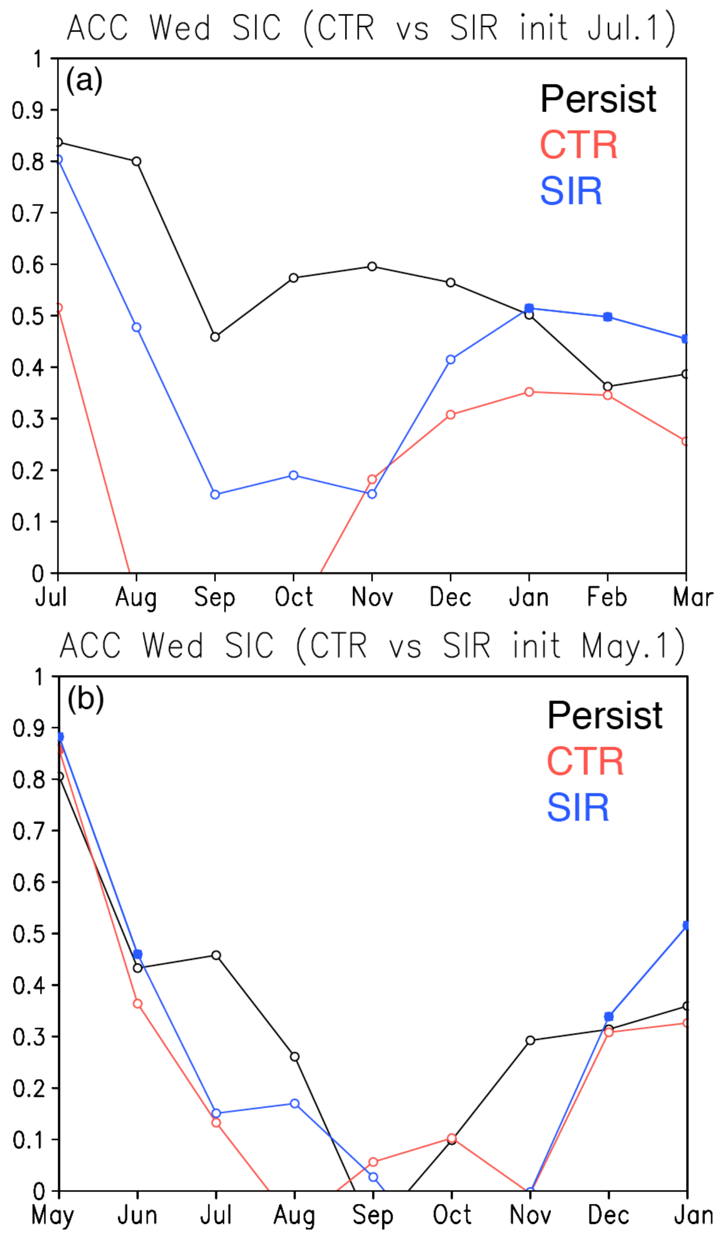

Figure S8: (a) Time series of anomaly correlation coefficients (ACC) of the observed sea-ice concentration (SIC) between June and subsequent months (Persistence; black line). Red (blue) line shows time series of the ACC between the observation and the CTR (SIR) reforecast results from July 1st. Solid circles on the CTR and SIR lines indicate the ACCs which exceed the persistence values and are statistically significant at 95% confidence level of a two-tailed Student's *t*-test. (b) Same as in (a), but for the case of the CTR (SIR) reforecast results from May 1st. The maps were generated using Grid Analysis and Display System (GrADS) Version 2.1.a3 (http://cola.gmu.edu/grads/downloads.php).

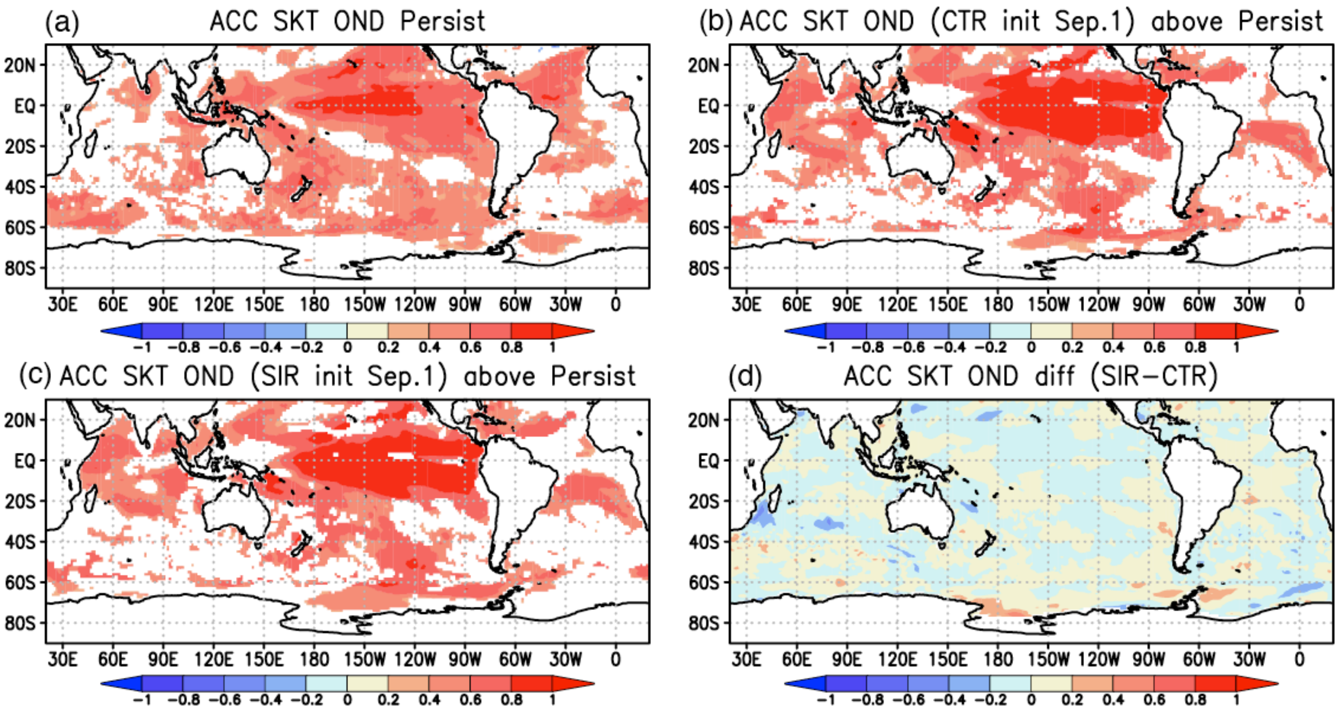

Figure S9: (a) Anomaly correlation coefficient (ACC; Persistence) of the observed surface air temperature (SAT) anomalies between Jun-Aug and Oct-Dec seasons. The statistically significant ACCs at 95% confidence level of a two-tailed Student's *t*-test are shaded. (b) ACC of the Oct-Dec mean SAT anomalies between the observation and the CTR reforecast results from September 1st. Positive ACCs which exceed persistence values in (a) and are statistically significant at 95% confidence level of a two-tailed Student's *t*-test are shaded. (c) Same as in (b), but from the SIR reforecast results from September 1st. (d) Differences in the ACCs between the SIR and CTR reforecast results. The maps were generated using Grid Analysis and Display System (GrADS) Version 2.1.a3 (http://cola.gmu.edu/grads/downloads.php).

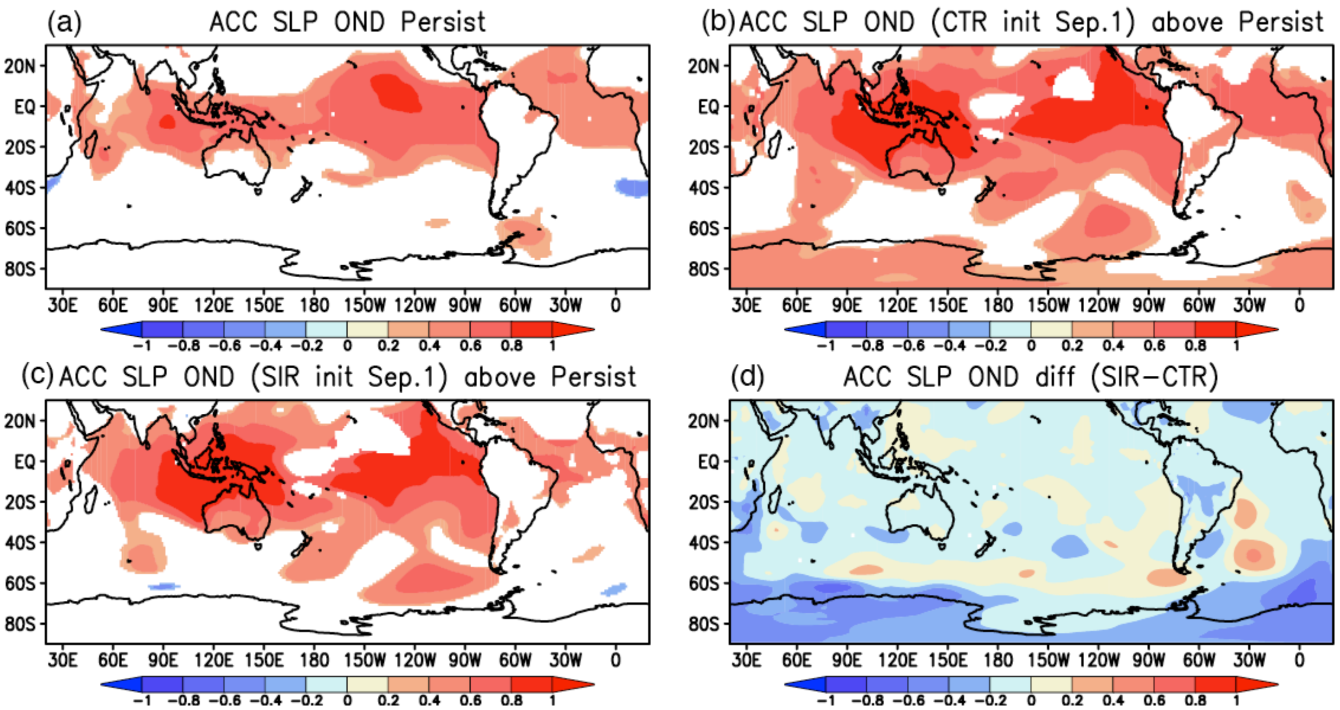

Figure S10: Same as in Fig. S9, but for the sea-level pressure (SLP) anomalies. The maps were generated using Grid Analysis and Display System (GrADS) Version 2.1.a3 (http://cola.gmu.edu/grads/downloads.php).
